# Modifying exercise and physical therapy in medical rehabilitation to promote a physically active lifestyle: Protocol for a multicenter controlled hybrid type 2 effectiveness-implementation study in three German rehabilitation centers

**DOI:** 10.1186/s13102-025-01179-2

**Published:** 2025-05-28

**Authors:** Eva Grüne, Johannes Carl, Wolfgang Geidl, Johanna Popp, David Victor Fiedler, Natalie Boss, Regina Brinkmann, Juliane von dem Bussche, Gabi Eichner, Kathrin Kleinert, Christoph Löhr, Florian Mex, Paul Schwanitz von Keitz, Till Richter, Joachim Schmidt, Rainer Tischendorf, Gorden Sudeck, Klaus Pfeifer

**Affiliations:** 1 Department of Sport Science and Sport, Friedrich-Alexander-Universität Erlangen-Nürnberg, Gebbertstr. 123b, D-91058 Erlangen, Germany; 2 Institute of Sports Science, University of Tübingen, Tübingen, Germany; 3 Institute for Physical Activity and Nutrition, Deakin University, Geelong, Australia; 4 Frankenland-Klinik, Bad Windsheim, Germany; 5 Rehafachzentrum Bad Füssing – Passau, Bad Füssing, Germany; 6 Orthopädische Klinik Tegernsee, Tegernsee, Germany

**Keywords:** Co-creation, Health promotion, Physical activity, Physical activity-related health competence, Physical literacy, Health literacy

## Abstract

**Introduction:**

Since rehabilitation programs often do not enhance long-term physical activity in persons with a health condition, there is a great need to arrange rehabilitation concepts towards a stronger emphasis on sustainable physical activity promotion. In this regard, the model of physical activity-related health competence (PAHCO) offers the potential for optimizing physical activity promotion. In the context of medical rehabilitation, the STABEKO study uses a co-creation approach to modify existing exercise and physical therapy according to PAHCO and long-term physical activity promotion in three rehabilitation centers in Bavaria, Germany. The objectives of the STABEKO study are A) to evaluate the implementation of modified exercise and physical therapy and to identify influencing factors for development and implementation, and B) to determine the short- and long-term effectiveness of the modified therapy on PAHCO and physical activity of the persons undergoing rehabilitation.

**Methods:**

As part of the STABEKO study, we will use cooperative planning as a co-creation approach for planning, developing, and implementing actions to modify exercise and physical therapy by engaging relevant actors from practice, policy, and research in an equal decision-making process. Within a multicenter controlled hybrid type 2 effectiveness-implementation study, we will simultaneously evaluate the effectiveness and implementation of modified exercise and physical therapy by collecting, analyzing, and triangulating qualitative and quantitative data at several measurement time points.

**Discussion:**

The STABEKO study will provide comprehensive insights into the implementation and effectiveness of exercise and physical therapy in medical rehabilitation, modified using a co-creation approach to promote long-term physical activity of persons undergoing rehabilitation. While the implementation study will indicate which modifications could be achieved and which factors have influenced their development and implementation, the effectiveness study will investigate whether modified exercise and physical therapy lead to changes in PAHCO and physical activity in persons undergoing rehabilitation. The results of this study will determine the dissemination of long-term physical activity promotion in the German rehabilitation sector and will provide important information for modifying existing therapeutic concepts to improve their effectiveness and implementation at an international level.

**Trial registration:**

The study was registered on the Open Science Framework on July 8 2024: 10.17605/OSF.IO/2N8UM and 10.17605/OSF.IO/J3KR2.

**Supplementary Information:**

The online version contains supplementary material available at 10.1186/s13102-025-01179-2.

## Introduction

Rehabilitation as a health strategy aims to optimize the functioning of people living with a health condition [1, 2]. This includes reducing the impact of health conditions on the daily lives of people affected and supporting them in remaining as independent as possible and participating in education, work, and meaningful life [3]. Behavioral change in physical activity (PA) is key to achieving these rehabilitation aims, as regular PA positively influences individuals’ exercise capacity and health-related quality of life for more than 25 health conditions [4]. Despite these benefits, individuals with a health condition show considerably lower PA levels compared to healthy adults [5, 6]. This makes exercise and physical therapy a central component of a rehabilitation program, especially as modern rehabilitation guidelines highlight the importance of physical activity promotion (PAP) [7–9]. However, increasing PA levels in people with health conditions is a major challenge, and sustainable changes toward a physically active lifestyle after rehabilitation are often rather limited [10].

**Fig. 1:**
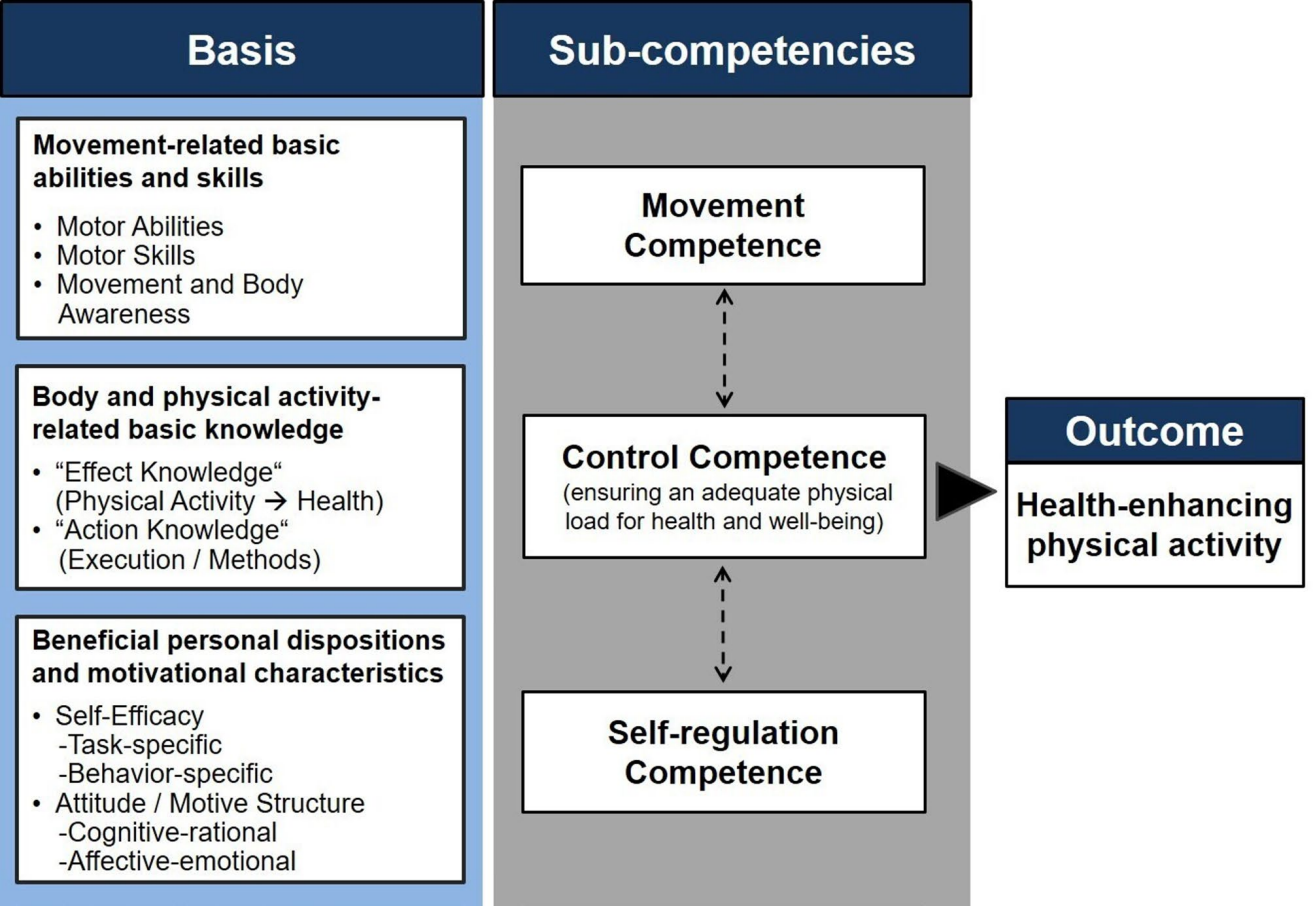
The physical activity-related health competence (PAHCO) model [11, 12]

The model of physical activity-related health competence (PAHCO) [11, 12] offers the potential for optimizing PAP in medical rehabilitation. The PAHCO model explicitly addresses “competencies” for physically active lifestyles, also in the (health) educational sense [13]. As such, the PAHCO model incorporates three major components, which are formed by the interplay of basic elements (Fig. 1). The framework posits that individuals require (i) movement competence, (ii) self-regulation competence, and (iii) control competence to execute PA in a health-oriented way. First, *movement competence* bundles the motor-related requirements to partake in planned, structured exercise (e.g., individual workout, leisure-time team sport) and to accomplish activities of daily living (e.g., cycling to work, gardening, household). Second, *self-regulation competence* ensures the regularity of PA (e.g., its frequent initiation and the duration that has to be maintained) by providing the necessary motivational and volitional resources (e.g., self-efficacy, PA-specific self-control). Third, *control competence* detaches from a mere orientation on quantity and guarantees that PA meet quality aspects by aligning with individual’s biopsychosocial health. This model component is based on aspects of knowledge, self-reflection, and understanding, which together ensure, for example, that physical stresses do not fall below or exceed thresholds of biopositive adaptation (i.e., physical health) and that activity modalities are selected that allow positive affective responses to PA (i.e., psychosocial health).

In summary, PAHCO overcomes the isolated functional orientation of PA by connecting “training and exercising” with “holistic individual learning” as well as “authentic experiencing” in contexts relevant for PA [11, 14]. Due to its long-term horizon, such a concept can make a pivotal contribution to sustainable rehabilitation success. In particular, the model can serve as a guide for exercise and physical therapy approaches with a focus on PAP. The model has found its way into the framework concepts of German rehabilitation standards [15]. However, recent analyses of the national rehabilitation landscape have illustrated that a minority of current exercise-related concepts and therapeutic practices within rehabilitation centers comply with the sustainable, empowering idea of PAHCO [16, 17]. The causes for this finding are manifold and mainly relate to the organizational-structural level (e.g., limited freedom for PA-promoting therapy due to rigid standard prescriptions, insufficient structures and processes for interprofessional collaboration) and the personal level (e.g., partly limited knowledge, skills, and abilities of therapists regarding PA-promoting therapy, low acceptance of PA-promoting therapy among persons undergoing rehabilitation) [18].

Accordingly, there is a great need to arrange rehabilitation concepts with a stronger emphasis on PAP. In this regard, the use of a co-creation approach appears promising, as the collaboration of researchers and non-academic actors facilitates the development and implementation of new actions that fit the setting and its individuals by taking into account the existing constraints and needs in such a way that these can be accepted and sustainably anchored within the system [19–21]. Several studies have demonstrated the successful application of co-creation approaches to improve health services and PAP in different settings [22–25], and the potential has also been addressed in the context of the rehabilitation system [26, 27]. Cooperative planning (CP) corresponds to such a co-creation approach and engages relevant actors from practice, policy, and research in an equal decision-making process to plan, develop, and implement actions to PAP [24, 28–31]. As a previous study from vocational education and training has shown that PAHCO, as a complex concept for PAP, was successfully adopted via CP [32], it appears worthwhile to use this approach to optimize PAP in medical rehabilitation. Therefore, the STABEKO study uses CP to modify exercise and physical therapy according to PAHCO and long-term PAP in three rehabilitation centers in Bavaria, Germany, to promote long-term PA in persons undergoing rehabilitation.

Following the structure of a type 2 hybrid effectiveness-implementation study [33, 34], the study “Partizipative Weiterentwicklung und Implementierung einer kompetenzorientierten Bewegungstherapie in der medizinischen Rehabilitation zur **Stä**rkung der **be**wegungsbezogenen Gesundheits**ko**mpetenz von Rehabilitand*innen” (STABEKO; Eng.: Participatory development and implementation of competence-oriented exercise and physical therapy in medical rehabilitation to strengthen PAHCO of persons undergoing rehabilitation) aims to simultaneously and equally evaluate the effectiveness and implementation of exercise and physical therapy in medical rehabilitation, modified by using CP. Specifically, the research objectives are as follows:

A) To evaluate the implementation of modified exercise and physical therapy achieved via CP and to identify influencing factors for development and implementation;

B) To determine the short- and long-term effectiveness of modified exercise and physical therapy achieved via CP on PAHCO and PA of persons undergoing rehabilitation.

## Methods

The present study protocol follows the Standard Protocol Items: Recommendations for Interventional Trials (SPIRIT) guidelines [35]. The SPIRIT checklist is provided in the supplementary material (see Supplementary Material 1). In combination with the present study protocol, the final reports will follow the Standards for Reporting Qualitative Research [36] for the implementation study, the 2010 Consort Statement [37] for the effectiveness study, and the Standards for Reporting Implementation Studies (StaRI) Statement [38] for the entire hybrid effectiveness-implementation study.

### Study design and setting

We will simultaneously evaluate the effectiveness and implementation of modified exercise and physical therapy within a multicenter non-randomized controlled trial. The study will take place in three inpatient rehabilitation centers in Bavaria, Germany, between September 2023 and May 2027. These rehabilitation centers are part of the clinic association of the German Pension Insurance (Ger.: Deutsche Rentenversicherung) in Bavaria, Germany, with a specialization in indications in the fields of orthopedics, rheumatology and/or internal medicine. According to the tenth revision of the International Statistical Classification of Diseases and Related Health Problems (ICD-10) [39], examples of health conditions treated in these clinics include type 2 diabetes mellitus (E11), obesity (E66), coxarthrosis (M16), gonarthrosis (M17), cervical disc disorders (M50) other intervertebral disc disorders (M51), other dorsopathies (M53), dorsalgia (M54), and shoulder lesions (M75). The rehabilitation centers confirmed their willingness to participate in this study in a letter of intent. The corresponding costs for the personnel resources required to complete the study are included in the funding requested and are provided to the rehabilitation centers.

### Study procedure

**Fig. 2:**
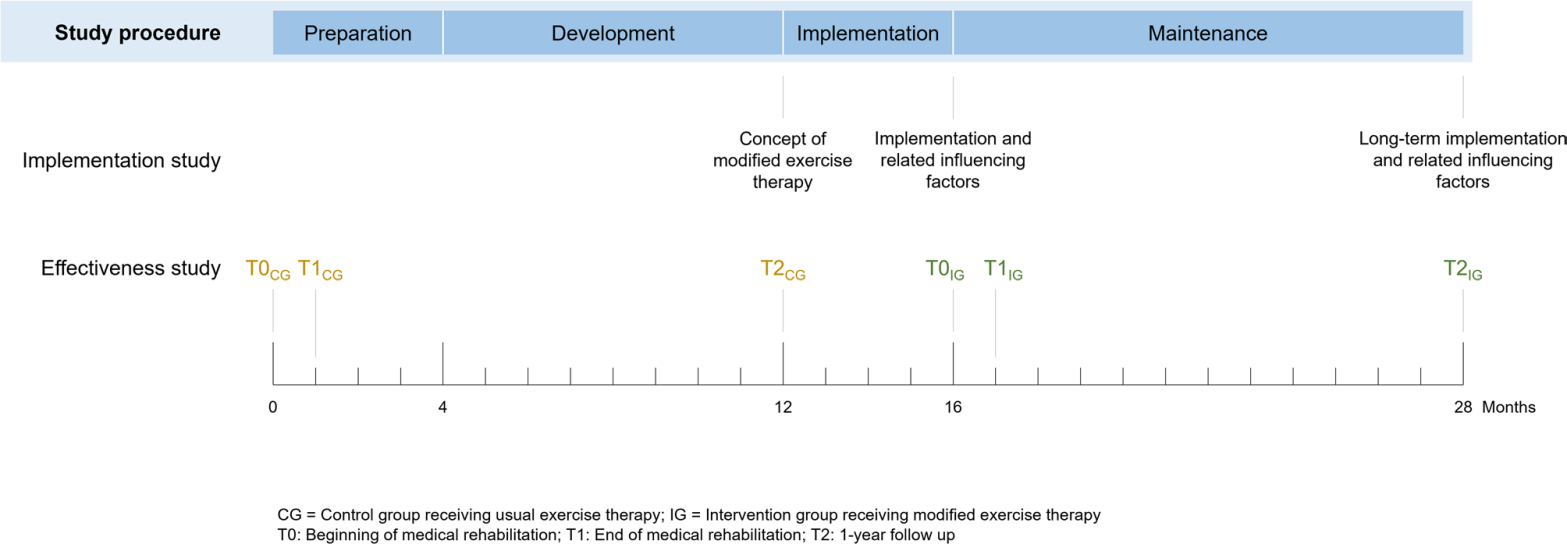
Overview of the study design

Within the STABEKO study, we will use CP as a co-creation approach to modify exercise and physical therapy in medical rehabilitation. More specifically, CP engages relevant actors from practice, policy, and research in an equal decision-making process to plan, develop, and implement actions to modify exercise and physical therapy [24, 28, 29, 31]. The study procedure will consist of the following four phases for each rehabilitation center: preparation phase, development phase, implementation phase, and maintenance phase (see Fig. 2).

The study procedure will start with a four-month *preparation phase*, in which the researchers familiarize themselves with the rehabilitation center, its organizational structures and processes, the persons undergoing rehabilitation, and identify relevant actors for joining a CP group. Therefore, initial meetings between actors from research, practice, and policy will take place, serving the presentation of the study procedure. In addition, a detailed explorative situation analysis will be carried out using mixed methods to obtain relevant information about the respective rehabilitation center (e.g., therapeutic concept, staff, organizational structures and processes) and its persons undergoing rehabilitation, which will be incorporated into the subsequent development phase.

The eight-month *development phase* is the centerpiece of CP. In this phase, four to six CP meetings will take place to jointly develop actions for modifying the existing exercise and physical therapy according to PAHCO and long-term PAP within the scope of a CP group. The CP group will consist of various actors from research (i.e., researchers), practice (e.g., exercise therapists, physical therapists, physicians, psychologists, social workers, administrative staff), and policy (e.g., clinic directors). While the researchers will provide the current scientific findings, moderate, and supervise the CP meetings, the actors from practice and policy will contribute their views and suggestions for modifying exercise and physical therapy and share their opinions on the opportunities for implementation. This phase will encompass the following steps in accordance with current recommendations for CP [28, 40]: (1) brainstorming of ideas and suggestions, (2) categorization of ideas and suggestions, (3) prioritization of suggestions, (4) definition of actions, (5) specification and finalization of actions, and (6) initiation. First, initial ideas for possible actions to modify exercise and physical therapy according to PAHCO and long-term PAP will be gathered based on the available scientific evidence and the results of the detailed explorative situation analysis. Potential starting points will be the therapy concept, human resource development, and organizational development. These ideas will subsequently be categorized and prioritized, taking into account their perceived attractiveness, relevance, and feasibility. In the following, concrete actions to modify exercise and physical therapy will be developed based on the prioritized ideas. These actions will be documented in an action plan that contains a detailed description of the content to be implemented along with the intended implementation, including the associated realization steps (e.g., the requirements to be fulfilled, the defined time frame and responsibilities). Finally, this action plan will be discussed, reflected, and finalized through repeated adjustments.

In the *implementation phase*, which lasts four months, the modified exercise and physical therapy will be implemented in accordance with the action plan under the responsibility of practitioners, with researchers providing advice and support. This will be followed by the *maintenance phase*, in which the modified exercise and physical therapy should be continued regularly and permanently.

Parallel to the abovementioned phases, we will evaluate implementation and effectiveness by collecting and analyzing qualitative and quantitative data at several measurement time points (for more details, see Implementation study or Effectiveness study). The study procedure will not be simultaneously launched in the participating rehabilitation centers; rather, the centers will be successively integrated with a 5-month delay to benefit from the experience previously gained. The STABEKO study will be monitored and supervised by an expert panel comprising various external actors (e.g., representatives of the German Pension Insurance [Ger.: Deutsche Rentenversicherung] in Bavaria, members of the exercise and physical therapy working group of the German Society for Rehabilitation Sciences [Ger.: Deutsche Gesellschaft für Rehabilitationswissenschaften e.V.], and representatives of the German Association for Health Sport and Sport Therapy [Ger.: Deutscher Verband für Gesundheitssport & Sporttherapie e.V.]).

### Implementation study

The implementation study aims to evaluate the implementation of modified exercise and physical therapy achieved via CP and to identify influencing factors for development and implementation by collecting, analyzing, and triangulating data following a mixed methods approach.

#### Outcomes

To evaluate the implementation of modified exercise and physical therapy, the modifications that were developed within the development phase and defined in the action plan (see Study procedure) will be examined. For this purpose, we will investigate the concept of modified exercise and physical therapy, the implementation status of the modifications, and patient-reported experience measures (PREMs). To evaluate the factors influencing the development and implementation of modified exercise and physical therapy, we will assess the CP meetings as well as the facilitators and barriers to development and implementation. Table 1 provides an overview of the evaluation outcomes, data sources, and measurement time points of the implementation study.

**Table 1:** Overview of data to be collected in the implementation study

Evaluation outcome		Data source		Measurement time points		
			Preparation phase	Development phase	Implementation phase	Maintenance phase
Implementation of modified exercise and physical therapy	Concept of modified exercise and physical therapy	Action plans			X	
	Implementation status of modifications	Self-developed questionnaire			X	X
	Patient-reported experience measures	Self-developed questionnaire	X			X
	Additional information on modified exercise and physical therapy	Semi-structured interviews			(X)	(X)
Influencing factors for development and implementation of modified exercise and physical therapy	Cooperative planning meetings	Structured minutes		X		
	Facilitators and barriers	Semi-structured interviews			X	X

#### Data collection

To evaluate the implementation of modified exercise and physical therapy, an initial document analysis of the action plan will be conducted in the implementation phase to gain an understanding of the concept of modified exercise and physical therapy (e.g., number and content of developed actions for modifications). Next, the implementation status of the modifications will be assessed in both the implementation and maintenance phases via a self-developed questionnaire. An exemplary question on the implementation status is: “To what extent has the modification been implemented as defined in the action plan?” Data will be collected analogously via a paper-pencil questionnaire. Following a purposeful sampling of information-rich cases [41], we will invite the main actors of each rehabilitation center to participate in this questionnaire survey in the respective implementation and maintenance phases. In addition, the PREMs will be conducted in the development phase and maintenance phase via a self-developed questionnaire to assess the experiences of the persons undergoing rehabilitation with exercise and physical therapy before and after the modification. An exemplary item of the PREMs, answered on a five-point Likert scale, is “During exercise and physical therapy, I received helpful information on how and where I can remain physically active independently after rehabilitation.” This questionnaire will be part of the effectiveness study’s data collection at the end of medical rehabilitation (T1) (see Effectiveness study).

To identify the factors influencing the development and implementation of modified exercise and physical therapy, structured minutes of all CP meetings will be taken during the development phase. These will contain information about the date and duration of the CP meetings, the participants, and the most important topics discussed. In addition, the facilitators and barriers for development and (long-term) implementation will be determined through semi-structured interviews. We will develop an interview guide based on the domains of the Consolidated Framework for Implementation Research (CFIR) [42, 43] and inspired by the explorative situation analysis conducted in the preparation phase. An exemplary question for identifying the influencing factors is “What contributed to the modification(s) being implemented / not being implemented?” In addition, the interviews will allow us to obtain additional information about the developed modifications, if required. In accordance with purposeful sampling of information-rich cases [41], we will again invite the main actors of each rehabilitation center (as described above for the questionnaire survey) to participate in an interview in the respective implementation and maintenance phases. One interview per participant will be conducted and audio-recorded either face-to-face, by telephone or video call.

#### Data management

The analog questionnaire data will be digitalized and then stored and processed in SPSS (IBM, Armonk, USA). The structured minutes will be saved on local drives. The audio-recorded interviews will be transcribed verbatim. For reasons of anonymity, we will replace personal names with professional titles as well as names of rehabilitation centers and cities with pseudonyms. The interview data will be stored and analyzed using MAXQDA (VERBI Software, Berlin).

#### Data analysis

The data collected in the course of the implementation study will be analyzed depending on the type of data source used. The document analyses of action plans and structured minutes will draw on a qualitative content analysis [44] and will be performed using MAXQDA (VERBI Software, Berlin). Data from the questionnaire survey on the implementation status and PREMs will be analyzed descriptively by using SPSS (IBM, Armonk, USA). The interview transcripts will be analyzed via qualitative content analysis [44], which includes a deductive and inductive definition of categories. This data analysis encompasses the following steps: (1) initial text work, (2) deductive development of the main categories based on the interview guide, (3) coding of the entire material according to the main categories, (4) compilation of all coded text passages of the same main category, (5) inductive definition of subcategories based on the transcribed material, (6) coding of the entire material according to the refined categories, and (7) evaluation and interpretation [44]. Two researchers will develop and double-check the main categories and subcategories and apply them to the interview transcripts. Inconsistencies will be discussed and resolved within the research team. MAXQDA (VERBI Software, Berlin) will be used for coding and analyzing the interview data.

Upon completion of the separate analyses, the data from the implementation study will be triangulated in the interpretation phase to provide a comprehensive description of the modifications developed and implemented and the factors influencing development and implementation [45]. In addition, the findings of the explorative situation analysis conducted in the preparation phase may be used to explore the modifications and influencing factors in more detail. Additionally, the results of the implementation study could be used to interpret the results of the effectiveness study.

### Effectiveness study

To determine the short- and long-term effectiveness of modified exercise and physical therapy on PAHCO and PA in persons undergoing rehabilitation, these effects will be compared with those of exercise and physical therapy conducted before modification (see Fig. 2). Owing to the prevailing organizational characteristics in the real-world setting, we will pursue a pragmatic evaluation approach based on a quasi-experimental design [46]. For this purpose, individuals undergoing inpatient medical rehabilitation will be recruited at two different time periods, namely, (a) prior to the development of modifications in the preparation phase, representing the control group (CG) receiving usual exercise and physical therapy, and (b) after the implementation of the modified exercise and physical therapy in the maintenance phase, representing the intervention group (IG) receiving modified exercise and physical therapy. In this context, we hypothesize that the changes in PAHCO and PA in persons undergoing rehabilitation after the modification of exercise and physical therapy (i.e., IG) will be significantly higher than the changes in PAHCO and PA in persons undergoing rehabilitation before the modification of exercise and physical therapy (i.e., CG).

#### Eligibility criteria

For inclusion in the study, the persons undergoing rehabilitation must fulfill the following eligibility criteria: (A) participation in an inpatient medical rehabilitation program at the respective time period (i.e., preparation phase for CG or maintenance phase for IG), (B) sufficient ability to read, comprehend, and write in the German language, and (C) provision of written informed consent to participate in the study. Accordingly, all participants who (A) discontinue the planned medical rehabilitation program, (B) have an insufficient ability to read, comprehend, and write in the German language, or (C) withdraw their original consent for participation are excluded.

#### Intervention

The participants in the CG will receive the usual exercise and physical therapy provided prior to the development and implementation of actions for modification. In Germany, a person undergoing rehabilitation usually receives approximately 11.8 h of physical and exercise therapy per week [47]. Exercise and physical therapy include services that use exercise and PA to achieve therapeutic goals (e.g., endurance, strength, and coordination training, treatments focusing on specific health conditions) that are organized either as individual or group sessions and delivered by qualified professionals (e.g., sports scientist, physiotherapists) [15]. The duration of the control period will correspond to the duration of the stay at the rehabilitation center, which is usually three weeks.

After the development and implementation of actions to modify exercise and physical therapy in the course of CP (see Study procedure), the participants in the IG will receive the modified exercise and physical therapy. Given the co-creation approach, the precise content of the modified exercise and physical therapy cannot yet be outlined in more detail. Similarly, the duration of intervention will equal the duration of the stay at the rehabilitation center (i.e., usually three weeks).

#### Outcomes

To evaluate whether the persons undergoing rehabilitation have acquired the competencies required to lead a healthy, physically active lifestyle, we will employ the PAHCO questionnaire with 42 items [48]. The assessment represents a self-report procedure and contains ten discriminant scales with theory-conform cross loadings across the three dimensions of movement competence, control competence, and self-regulation competence. The questionnaire has been validated in a stepwise manner across different target groups [14, 48]. Motivational competence for sport and exercise describes the self-determined ability to select convenient exercise and sport activities [49]. It has already been discussed as a motivational facet of PA-specific self-regulation competence within the original work on the PAHCO model [12], but an assessment instrument was only recently validated with a sample of adults [49]. Thus, we will complement the PAHCO assessment through the suggested scale with four items [49].

Among the secondary outcomes, we will operationalize the volume of PA by the two-dimensional “Bewegungs- und Sportaktivität” (BSA; Eng.: Physical Activity, Exercise, and Sport) questionnaire [50] which assesses leisure-time PA and sport activity over the past four weeks. The instrument specifies eight important activities of daily living (i.e., walking to work, walking to shopping, cycling to work, cycling for other reasons of transportation, taking a walk, gardening, strenuous housework, strenuous care work) for leisure-time PA and provides the opportunity to report three different, self-selected sport activities.

We will measure the general health status via the “Indikatoren des Reha-Status” (IRES; Eng.: Indicators of the Rehabilitation Status) questionnaire [51]. More specifically, we will use the IRES-24 questionnaire, which constitutes the short version of a comprehensive, 144 item self-report survey in the German language. The 24 items must be answered on either a five- or six-point Likert scale and encompass the sub-scales of subjective health, functionality in everyday life, physical health, and pain.

In addition, we will acquire basic sociodemographic data (e.g., age, sex, height, weight, employment status, and education), disease-specific information (e.g., indication/diagnosis, disease severity/degree of disability, comorbidities), and therapy-specific information (e.g., duration and content of exercise and physical therapy) of the persons undergoing rehabilitation. Primary and secondary outcomes are based on hypothesized intervention effects and are listed in Table 2.

**Table 2:** Overview of data to be collected in the effectiveness study

Construct	Assessment and reference	Measurement time points		
		Beginning of medical rehabilitation (T0)	End of medical rehabilitation (T1)	1-year follow-up (T2)
**Primary outcome: Physical activity-related health competence (PAHCO)**				
Movement competence	PAHCO Questionnaire: subscales of movement competence [48]	X	X	X
Control competence	PAHCO Questionnaire: subscales of control competence [48]	X	X	X
Self-regulation competence	PAHCO Questionnaire: subscales of self-regulation competence [48]	X	X	X
	Questionnaire on motivational competence in exercise and sport [49]	X	X	X
**Secondary outcomes**				
Physical activity	“Bewegungs- und Sportaktivität” (BSA; Eng.: Physical Activity, Exercise, and Sport) questionnaire (with the dimensions leisure-time PA and sport activity) [50]	X		X
General health	Short version of the “Indikatoren des Reha-Status” (IRES; Eng.: Indicators of the Rehabilitation Status) questionnaire [51]	X	X	X
**Further participant data**				
Basic sociodemographic data	Questionnaire on age, height, weight, sex, work ability, employment status, education	X		
Disease-specific information^1^	Medical form stating indication/diagnosis, disease severity/degree of disability, comorbidities	X		
Therapy-specific information^1^	Documentation form stating duration and content of exercise and physical therapy		X	

Note: ^1^We cannot assume uniform assessments for these data, as the assessments may vary between the rehabilitation centers, for example due to different standard diagnostics or therapy documentation

#### Sample size

With the three PAHCO dimensions (i.e., movement competence, control competence, and self-regulation competence), the present study lists three main outcomes. We set *p* ≤.05 for the repeated multivariate analyses of covariance (MANCOVA) and assume a pre-post correlation (within-subjects factor) of *r* = .40, as a previous study revealed an autoregressive *r* = .418 [52]. To detect a significant intervention effect of medium magnitude (*η*^2^ = 0.04; *f* = 0.20) paired with a statistical power ≥ 80%, we must recruit a total of 244 persons undergoing rehabilitation. Given the number of rehabilitation centers (*n* = 3), we are advised to aim for 82 participants per center. Since each rehabilitation center includes persons undergoing rehabilitation at two time periods, that is, before modification (i.e., CG) and after modification (i.e., IG), we must recruit a number of *n* = 41 participants per time period in the respective rehabilitation center. Considering a longitudinal dropout rate of 15%, we aim to recruit *n* = 48 participants per time period in each rehabilitation center.

#### Recruitment

The recruitment of participants will be conducted by a study coordinator who is employed by the rehabilitation center. This study coordinator will screen all persons undergoing rehabilitation, who start their medical rehabilitation program during either the preparation phase (i.e., CG) or maintenance phase (i.e., IG), for their eligibility.

#### Blinding

As all participants will receive both verbal and written information about the study as the legal basis for their informed consent to participate, they will be aware of the scientific purpose of evaluating exercise and physical therapy in medical rehabilitation. However, concealment can be ensured as to whether participants are part of the IG or CG. In contrast, exercise and physical therapists, as providers of the intervention, will be fully aware of the study procedure, meaning that no blinding can be realized at the facilitator level.

#### Data collection

To evaluate the effectiveness of the modified exercise and physical therapy in the short- and long-term, outcomes will be assessed at the beginning of medical rehabilitation (T0), at the end of medical rehabilitation (T1), and one year after the beginning of medical rehabilitation (T2) (see Fig. 2). We will collect participants’ data on PAHCO, PA, general health and basic socio-demographic information via a paper-pencil questionnaire. The disease-specific information will be obtained via a medical form, which will be used as part of routine diagnostics at the participating rehabilitation centers, and the therapy-specific information will be gathered via a documentation form, which will be completed by the study coordinator. The study coordinator located at each rehabilitation center will administer the data collection procedure by handing out the questionnaires at the beginning (T0) and end (T1) of medical rehabilitation and sending them via postal mail at the 1-year follow up (T2).

#### Data management

In addition to the self-reported data of the persons undergoing rehabilitation, the study coordinator will digitalize the disease-specific information from the medical form. A continuous pseudonym will link all the data sources of the participants across the time points. The storage and processing of the data collected will be performed in SPSS (IBM, Armonk, USA).

#### Data analysis

The data set for persons undergoing rehabilitation will contain longitudinal data at the individual level. Specifiable as within-subjects factors, calculations can encompass comparisons between the beginning (T0) and the end of medical rehabilitation (T1) as short-term effects and respective comparisons with data at one year after starting medical rehabilitation (T2) as follow-up effects. In addition, the data set will contain longitudinal data on the level of the rehabilitation center (i.e., prior to the development of modifications, representing the CG, and after the implementation of the modified exercise and physical therapy, representing the IG; between-subjects factor). Since the effectiveness may vary among the three rehabilitation centers, we consider the setting as a control variate. We will integrate all these factors into repeated MANCOVA with the primary outcomes. To counteract outlier problems with the PA outcome, we will apply the winsorization technique to cut all values beyond the 95th percentile [53]. We will subsequently use the indicated frequencies and mean durations to calculate a corresponding total index in addition to a separate sport activity index. Calculations with the secondary outcomes will be treated as exploratory analyses. Nevertheless, we will adopt an intention-to-treat paradigm, meaning that all persons undergoing rehabilitation meeting the eligibility criteria and initially providing consent will be included in the statistical effectiveness computations. Reasonable dropouts (e.g., discontinuation of the planned medical rehabilitation program) will be treated as structurally missing data, whereas other missing items and measurements will be imputed with the expectation maximization algorithm [54]. We will run the analyses with the open source R (version 4.3.1 or higher) and interpret effect sizes (*η*²) according to the guidelines by Cohen [55], providing benchmarks to define small (*η*^2^ = 0.01), medium (*η*^2^ = 0.06), and large (*η*^2^ = 0.14) effects.

### Dissemination

In addition to the study procedure outlined previously, a scaling-up phase is intended in which researchers, in collaboration with further actors from policy and practice, develop a strategy for disseminating exercise and physical therapy focused on PAP at the regional or national level based on the results of the implementation and effectiveness study. This strategy will be developed based on an appropriate concept for dissemination or scaling-up (e.g., PRACTical planning for Implementation and Scale-up [56], ExpandNet/WHO conceptual framework for scaling-up [57]).

The study results will be published in peer-reviewed journals and presented at national and international scientific conferences.

## Discussion

Current practices in rehabilitation are insufficiently successful in preparing individuals for a self-organized physically active lifestyle. Previous studies, including those from the specific German context, have shown that the majority of concepts in exercise and physical therapy focus overly on functional aspects instead of comprehensively addressing the breadth of modifiable personal determinants of health-enhancing PA behavior [16–18]. In this regard, the present study will apply a co-creation approach to revise and ideally improve current PAP concepts within three German rehabilitation centers. With the structure of a type 2 hybrid effectiveness-implementation study, this study will provide comprehensive insights into the implementation and effectiveness of modified exercise and physical therapy in medical rehabilitation. More specifically, the implementation study will indicate which modifications could be achieved and which factors have influenced their development and implementation, whereas the effectiveness study will investigate whether modified exercise and physical therapy lead to improvements in PAHCO and PA among persons undergoing rehabilitation.

Although the present study may add value to PAP within medical rehabilitation, we consider the following limitations. First, all rehabilitation centers are longitudinally compared against their previous concept (i.e., intra-organizational control with intervention and control periods). Against this background, we cannot fully exclude that cohort or historical effects over time (e.g., reforms of the entire rehabilitation system, severe disease waves, labor disputes of therapist associations) may affect the results, which cannot be methodologically controlled by this study design. In any case, we will closely follow relevant processes within the rehabilitation centers (e.g., other interventions being conducted in parallel with our study) as well as the political atmosphere, health-related debates, and upcoming guidelines relevant for the German rehabilitation system (e.g., via the expert panel) to provide potential explanations for the final interpretation of the results. Furthermore, the study procedure causes a 16-month time gap between the measurement time points of the CG and the IG, so that potential seasonal effects cannot be eliminated. However, the seasonal effects across the three rehabilitation centers are not systematically distributed for either the IG or the CG because of the sequential design and the associated measurement time points. Second, to the best of our knowledge, this study is the first to apply a co-creation approach to modify exercise and physical therapy in medical rehabilitation, with the aim of promoting long-term PA among persons undergoing rehabilitation in Germany. Owing to the complex approach to modifying the therapy concept, the study can only include three rehabilitation centers. Accordingly, we can only treat the rehabilitation centers as covariates instead of systematically considering the cluster effect through multilevel modeling (for sample requirements, see [58]).

Nevertheless, this study could serve as a starting point for promoting long-term health-enhancing PA in the context of medical rehabilitation. The evaluation will provide empirical arguments as to whether and how such a co-creation approach can contribute to the promotion of a healthy, physically active lifestyle among inpatient persons undergoing rehabilitation. Thus, the findings of this study will not only determine the dissemination of PAHCO in the German rehabilitation sector but will also provide important information for the modification of current exercise and physical therapy concepts to improve their effectiveness and implementation at an international level.

## Electronic supplementary material

Below is the link to the electronic supplementary material.

Supplementary Material 1

## Data Availability

No datasets were generated or analysed during the current study.

## Declarations

### Trial Status

This article represents the revised version of the study protocol. The study was registered online on the Open Science Framework on July 8, 2024: 10.17605/OSF.IO/2N8UM and 10.17605/OSF.IO/J3KR2. The recruitment of participants has started on February 15, 2024 (i.e., beginning of T0 in the first rehabilitation center) and is expected to end in March 2026 (i.e., completion of T0 in the third rehabilitation center).

## Abbreviations

BSA: Bewegungs- und Sportaktivität (Eng.: Physical Activity Exercise and Sport)
CFIR: Consolidated Framework for Implementation Research
CG: Control group
CP: Cooperative planning
IG: Intervention group
ICD-10: International Statistical Classification of Diseases and Related Health Problems, 10th revision
IRES: Indikatoren des Reha-Status (Eng.: Indicators of the Rehabilitation Status)
MANCOVA: Multivariate analyses of covariance
PA: Physical activity
PAHCO: Physical activity-related health competence
PAP: Physical activity promotion
PREMs: Patient-reported experience measures
STABEKO: Partizipative Weiterentwicklung und Implementierung einer kompetenzorientierten Bewegungstherapie in der medizinischen Rehabilitation zur Stärkung der bewegungsbezogenen Gesundheitskompetenz von Rehabilitand*innen (Eng.: Participatory development and implementation of competence-orientated exercise and physical therapy in medical rehabilitation to strengthen physical activity-related health competence among persons undergoing rehabilitation)

## References

[1] Meyer T, Gutenbrunner C, Bickenbach J, Cieza A, Melvin J, Stucki G. Towards a conceptual description of rehabilitation as a health strategy. JRM. 2011;43:765–9. 10.2340/16501977-0865. 21826389 10.2340/16501977-0865

[2] Stucki G, Bickenbach J, Gutenbrunner C, Melvin J, Rehabilitation. The health strategy of the 21st century. JRM. 2018;50:309–16. 10.2340/16501977-2200. 28140419 10.2340/16501977-2200

[3] World Health Organization. Rehabilitation. Fact sheet. 2023. https://www.who.int/news-room/fact-sheets/detail/rehabilitation. Accessed 23 Feb 2024.

[4] Dibben GO, Gardiner L, Young HM, Wells V, Evans RA, Ahmed Z, et al. Evidence for exercise-based interventions across 45 different long-term conditions: an overview of systematic reviews. eClinicalMedicine. 2024;102599. 10.1016/j.eclinm.2024.102599. 10.1016/j.eclinm.2024.102599 PMC11247153 39010975

[5] Sudeck G, Geidl W, Abu-Omar K, Finger JD, Krauß I, Pfeifer K. Do adults with non-communicable diseases Meet the German physical activity recommendations? Ger J Exerc Sport Res. 2021;51:183–93. 10.1007/s12662-021-00711-z.

[6] Brawner CA, Churilla JR, Keteyian SJ. Prevalence of physical activity is lower among individuals with chronic disease. Med Sci Sports Exerc. 2016;48:1062–7. 10.1249/MSS.0000000000000861. 26741117 10.1249/MSS.0000000000000861

[7] World Health Organization. Package of interventions for rehabilitation. Module 4. Cardiopulmonary conditions. 2023. https://www.who.int/publications/i/item/9789240071162. Accessed 23 Feb 2024.

[8] World Health Organization. Package of interventions for Rehabilitation. Module 2. Musculosceletal conditions. 2023. Package of interventions for Rehabilitation. Module 2. Musculosceletal conditions. Accessed 23 Feb 2024.

[9] Spruit MA, Singh SJ, Garvey C, ZuWallack R, Nici L, Rochester C, et al. An official American thoracic society/European respiratory society statement: key concepts and advances in pulmonary rehabilitation. Am J Respir Crit Care Med. 2013;188:e13–64. 10.1164/rccm.201309-1634ST. 24127811 10.1164/rccm.201309-1634ST

[10] Karloh M, Matias TS, de Oliveira JM, de Lima FF, Araújo Pinheiro DH, Barbosa GB, et al. Breaking barriers to rehabilitation: the role of behavior change theories in overcoming the challenge of exercise-related behavior change. Braz J Phys Ther. 2023;27:100574. 10.1016/j.bjpt.2023.100574. 38056192 10.1016/j.bjpt.2023.100574 PMC10749239

[11] Carl J, Sudeck G, Pfeifer K. Competencies for a healthy physically active lifestyle – Reflections on the model of physical Activity-related health competence (PAHCO). J Phys Activity Health. 2020. 10.1123/jpah.2019-0442. 10.1123/jpah.2019-0442 32473589

[12] Sudeck G, Pfeifer K. Physical activity-related health competence as an integrative objective in exercise therapy and health sports – conception and validation of a short questionnaire. German J Exerc Sport Res. 2016;46:74–87. 10.1007/s12662-016-0405-4.

[13] Klieme E, Hartig J, Rauch D. The concept of competence. In: Hartig J, Klieme E, Leutner D, editors. The concept of competence in educational contexts. Göttingen: Hogrefe & Huber; 2010. pp. 3–22.

[14] Sudeck G, Rosenstiel S, Carl J, Pfeifer K. Bewegungsbezogene Gesundheitskompetenz – Konzeption und Anwendung in gesundheitsförderung, Prävention und rehabilitation [Physical activity-related health competence - conception and application in health promotion, prevention, and rehabilitation]. In: Rathmann K, Dadaczynski K, Okan O, Messer M, editors. Gesundheitskompetenz. Berlin, Heidelberg: Springer Berlin Heidelberg; 2022. pp. 1–12. 10.1007/978-3-662-62800-3_135-1.

[15] Deutsche Rentenversicherung Bund. KTL. Klassifikation therapeutischer leistungen in der medizinischen Rehabilitation - Version 2015. Buck: Berlin; 2014.

[16] Deprins J, Geidl W, Streber R, Pfeifer K, Sudeck G. Konzeptionelle grundlagen der bewegungstherapie in der medizinischen rehabilitation: ergebnisse einer bundesweiten Bestandsaufnahme. Rehabilitation (Stuttg). 2019;58:366–75. 30677781 10.1055/a-0808-0814

[17] Sudeck G, Geidl W, Deprins J, Pfeifer K. The role of physical activity promotion in typical exercise therapy concepts: a latent class analysis based on a National survey in German rehabilitation settings. Disabil Rehabil. 2019;57:1–11. 10.1080/09638288.2019.1608322. 10.1080/09638288.2019.1608322 31079505

[18] Geidl W, Sudeck G, Wais J, Pfeifer K. Bewegungsförderliche bewegungstherapie in der medizinischen rehabilitation: Konsequenzen der bundesweiten Bestandsaufnahme für die qualitätsentwicklung. Rehabilitation (Stuttg). 2022;61:336–43. 34933356 10.1055/a-1693-8380

[19] Leask CF, Sandlund M, Skelton DA, Altenburg TM, Cardon G, Chinapaw MJM, et al. Framework, principles and recommendations for utilising participatory methodologies in the co-creation and evaluation of public health interventions. Res Involv Engagem. 2019;5:1–16. 30652027 10.1186/s40900-018-0136-9 PMC6327557

[20] Leeuw Ed, McNess A, Crisp B, Stagnitti K. Theoretical reflections on the nexus between research, policy and practice. 0958–1596. 2008.

[21] Vargas C, Whelan J, Brimblecombe J, Allender S. Co-creation, co-design, co-production for public health: a perspective on definition and distinctions. Public Health Res Pract. 2022;32. 10.17061/phrp3222211 35702744

[22] Halvorsrud K, Kucharska J, Adlington K, Rüdell K, Brown Hajdukova E, Nazroo J, et al. Identifying evidence of effectiveness in the co-creation of research: a systematic review and meta-analysis of the international healthcare literature. J Public Health. 2021;43:197–208. 10.1093/pubmed/fdz126 PMC8042368 31608396

[23] Barber R, Beresford P, Boote J, Cooper C, Faulkner A. Evaluating the impact of service user involvement on research: a prospective case study. Int J Consumer Stud. 2011;35:609–15.

[24] Gelius PC, Jansen M, King AC. Cooperative planning and its utilization in German physical activity promotion: a brief introduction. Health Promot Int. 2021;36:ii1–7. 10.1093/heapro/daab170. 34905606 10.1093/heapro/daab170 PMC8670624

[25] Smith B, Williams O, Bone L, Collective tMSWC. Co-production: A resource to guide co-producing research in the sport, exercise, and health sciences. Qualitative Res Sport Exerc Health. 2023;15:159–87. 10.1080/2159676X.2022.2052946.

[26] Hudon A, Gervais M-J, Hunt M. The contribution of conceptual frameworks to knowledge translation interventions in physical therapy. Phys Ther. 2015;95:630–9. 25060959 10.2522/ptj.20130483 PMC4384052

[27] Cardoso RS, Silva Filho LV. The co-production of innovation: A case study in a rehabilitation hospital. RAM. Revista de Administração Mackenzie. 2016;17:109–29. 10.1590/1678-69712016/administracao.v17n4p108-129

[28] Rütten A, Semrau J, Wolff AR. Entwicklung gesundheitsförderlicher strukturen durch kooperative Planung. Präv Gesundheitsf. 2023. 10.1007/s11553-023-01045-4.

[29] Rütten A. Kooperative Planung und gesundheitsförderung: Ein Implementationsansatz [Cooperative Planning and Health Promotion: An Implementation Approach]. J Public Health. 1997;5.

[30] Krause C, Sommerhalder K, Beer-Borst S, Abel T. Just a subtle difference? Findings from a systematic review on definitions of nutrition literacy and food literacy. Health Promot Int. 2016;10:daw084. 10.1093/heapro/daw084. 10.1093/heapro/daw084 PMC6005107 27803197

[31] Sommer R, Linder S, Ziemainz H, Gelius P. Key performance indicators of cooperative planning processes: case study results from German sport science and physical activity promotion projects. Ger J Exerc Sport Res. 2022;52:24–38. 10.1007/s12662-021-00745-3.

[32] Popp J, Grüne E, Carl J, Semrau J, Pfeifer K. Co-creating physical activity interventions: findings from a multiple case study using mixed methods. Front Public Health. 2022;10:975638. 36211644 10.3389/fpubh.2022.975638 PMC9534180

[33] Curran GM, Bauer M, Mittman B, Pyne JM, Stetler C. Effectiveness-implementation hybrid designs: combining elements of clinical effectiveness and implementation research to enhance public health impact. Med Care. 2012;50:217. 22310560 10.1097/MLR.0b013e3182408812 PMC3731143

[34] Curran GM, Landes SJ, McBain SA, Pyne JM, Smith JD, Fernandez ME, et al. Reflections on 10 years of effectiveness-implementation hybrid studies. Front Health Serv. 2022;2:1053496. 10.3389/frhs.2022.1053496. 36925811 10.3389/frhs.2022.1053496 PMC10012680

[35] Chan A-W, Tetzlaff JM, Gøtzsche PC, Altman DG, Mann H, Berlin JA, et al. SPIRIT 2013 explanation and elaboration: guidance for protocols of clinical trials. BMJ. 2013;346:e7586. 10.1136/bmj.e7586. 23303884 10.1136/bmj.e7586 PMC3541470

[36] O’Brien BC, Harris IB, Beckman TJ, Reed DA, Cook DA. Standards for reporting qualitative research: a synthesis of recommendations. Acad Med. 2014;89:1245–51. 24979285 10.1097/ACM.0000000000000388

[37] Schulz KF, Altman DG, Moher D. CONSORT 2010 statement: updated guidelines for reporting parallel group randomised trials. J Pharmacol Pharmacotherapeutics. 2010;1:100–7. 10.4103/0976-500X.72352 PMC3043330 21350618

[38] Pinnock H, Barwick M, Carpenter CR, Eldridge S, Grandes G, Griffiths CJ, et al. Standards for reporting implementation studies (StaRI): explanation and elaboration document. BMJ Open. 2017;7:e013318. 10.1136/bmjopen-2016-013318. 28373250 10.1136/bmjopen-2016-013318 PMC5387970

[39] World Health Organization. International statistical classification of diseases and related health problems: ICD-10. 10th ed. Geneva: World Health Organization; 2016.

[40] Popp J, Carl J, Grüne E, Semrau J, Gelius P, Pfeifer K. Physical activity promotion in German vocational education: does capacity Building work? Health Promot Int. 2020;1–13. 10.1093/heapro/daaa014. 10.1093/heapro/daaa014 PMC7785309 32105312

[41] Patton MQ. Qualitative research and evaluation methods: integrating theory and practice. 4th ed. Los Angeles: SAGE; 2015.

[42] Damschroder LJ, Aron DC, Keith RE, Kirsh SR, Alexander JA, Lowery JC. Fostering implementation of health services research findings into practice: a consolidated framework for advancing implementation science. Implement Sci. 2009;4:50. 10.1186/1748-5908-4-50. 19664226 10.1186/1748-5908-4-50 PMC2736161

[43] Damschroder LJ, Reardon CM, Widerquist MAO, Lowery J. The updated consolidated framework for implementation research based on user feedback. Implement Sci. 2022;17:75. 10.1186/s13012-022-01245-0. 36309746 10.1186/s13012-022-01245-0 PMC9617234

[44] Kuckartz U, Rädiker S. Qualitative inhaltsanalyse. methoden, praxis, Computerunterstützung: grundlagentexte methoden. 5th ed. Weinheim, Basel: Beltz Juventa; 2022.

[45] O’Cathain A, Murphy E, Nicholl J. Three techniques for integrating data in mixed methods studies. BMJ. 2010;341:c4587. 10.1136/bmj.c4587. 20851841 10.1136/bmj.c4587

[46] Crane M, Bauman A, Lloyd B, McGill B, Rissel C, Grunseit A. Applying pragmatic approaches to complex program evaluation: A case study of implementation of the new South Wales get healthy at work program. Health Promot J Austr. 2019;30:422–32. 10.1002/hpja.239. 30860630 10.1002/hpja.239

[47] Brüggemann S, Sewöster D, Kranzmann A. Bewegungstherapeutische versorgung in der medizinischen rehabilitation der Rentenversicherung – eine analyse auf basis quantitativer routinedaten [Exercise Therapy in German Medical rehabilitation – an Analysis based on Quantitative Routine Data]. [Exercise Therapy in German Medical rehabilitation – an Analysis based on Quantitative Routine Data]. Rehabilitation (Stuttg). 2018;57:24–30. 10.1055/s-0043-102556. 28746951 10.1055/s-0043-102556

[48] Carl J, Sudeck G, Pfeifer K. Competencies for a healthy physically active lifestyle – Second-order analysis and multidimensional scaling. Frontiers in Psychology. 2020;11:Article 558850. 10.3389/fpsyg.2020.558850 10.3389/fpsyg.2020.558850 PMC7779792 33408660

[49] Schorno N, Sudeck G, Gut V, Conzelmann A, Schmid J. Choosing an activity that suits: development and validation of a questionnaire on motivational competence in exercise and sport. Ger J Exerc Sport Res. 2021;51:71–8.

[50] Fuchs R, Klaperski S, Gerber M, Seelig H. Messung der Bewegungs- und sportaktivität Mit dem BSA-Fragebogen [Measurement of Physical Activity and Sport Activity With the BSA Questionnaire]. Z Für Gesundheitspsychologie. 2015;23:60–76. 10.1026/0943-8149/a000137.

[51] Wirtz M, Farin E, Bengel J, Jäckel WH, Hämmerer D, Gerdes N. IRES-24 patientenfragebogen. Diagnostica. 2005;51:75–87.

[52] Carl JA, Geidl W, Schuler M, Mino E, Lehbert N, Wittmann M, et al. Towards a better Understanding of physical activity in people with COPD: predicting physical activity after pulmonary rehabilitation using an integrative competence model. Chron Respir Dis. 2021;18:1479973121994781. 10.1177/1479973121994781. 33703932 10.1177/1479973121994781 PMC8718156

[53] Dixon WJ, Tukey JW. Approximate behavior of the distribution of winsorized t (Trimming/Winsorization 2). Technometrics. 1968;10:83–98.

[54] Lüdtke O, Robitzsch A, Trautwein U, Köller O. Umgang Mit Fehlenden werten in der Psychologischen forschung [Handling of missing data in psychological research: Problems and solutions]. Psychologische Rundschau. 2007;58:103–17. 10.1026/0033-3042.58.2.103.

[55] Cohen J. Statistical power analysis for the behavioral sciences. 2nd ed. Hillsdale, NJ: Lawrence Erlbaum Associates; 1988.

[56] Koorts H, Eakin E, Estabrooks P, Timperio A, Salmon J, Bauman A. Implementation and scale up of population physical activity interventions for clinical and community settings: the PRACTIS guide. Int J Behav Nutr Phys Act. 2018;15:51. 10.1186/s12966-018-0678-0. 29884236 10.1186/s12966-018-0678-0 PMC5994105

[57] World Health Organization. ExpandNet. Nine steps for developing a scaling-up strategy. Geneva: World Health Organization; 2010.

[58] McNeish D. Small sample methods for multilevel modeling: A colloquial Elucidation of REML and the Kenward-Roger correction. Multivar Behav Res. 2017;52:661–70. 10.1080/00273171.2017.1344538. 10.1080/00273171.2017.1344538 28715244

